# Effects of irrigation water salinity on evapotranspiration modified by leaching fractions in hot pepper plants

**DOI:** 10.1038/s41598-017-07743-2

**Published:** 2017-08-03

**Authors:** Rangjian Qiu, Chunwei Liu, Zhenchang Wang, Zaiqiang Yang, Yuanshu Jing

**Affiliations:** 1 Collaborative Innovation Center on Forecast and Evaluation of Meteorological Disasters, Jiangsu Key Laboratory of Agricultural Meteorology, Nanjing University of Information Science and Technology, Nanjing, 210044 China; 2 College of Water Conservancy and Hydropower Engineering, Hohai University, Nanjing, 210098 China

## Abstract

We investigated whether leaching fraction (LF) is able to modify the effects of irrigation water salinity (EC_iw_) on evapotranspiration (ET). We conducted an experiment with a completely randomized block design using five levels of EC_iw_ and two LFs. Results showed that the electrical conductivity of drainage water (EC_dw_) in an LF of 0.29 was considerably higher during the 21–36 days after transplanting (DAT), and considerably lower after 50 DAT than in an LF of 0.17. The hourly, nighttime, daily, cumulative and seasonal ET all decreased considerably as a result of an increase in the EC_iw_. The daily ET started to be considerably higher in the LF of 0.29 than in the LF of 0.17 from 65 DAT. Compared with the LF of 0.17, the seasonal ET in the LF of 0.29 under various EC_iw_ levels increased by 4.8%–8.7%. The Maas and Hoffman and van Genuchten and Hoffman models both corresponded well with the measured relative seasonal ET and the LF had no marked effects on these model parameters. Collectively, an increase in the level of EC_iw_ always decreased the ET substantially. An increase in the LF increased the ET considerably, but there was a time lag.

## Introduction

Evapotranspiration (ET), causing the movement of water, nutrients and minerals from the roots to the plant organs, plays an important role in growth and water productivity. ET is affected by many factors, for example weather, crop factors, management and environment^1^. Salinity is one of main factors affecting ET. Previous studies have shown that an increase in irrigation water salinity (EC_iw_) leads to a decrease in transpiration, resulting in reduced ET. A linear reduction in ET has been observed for several crop types with varying levels of EC_iw_, including bell peppers (*Capsicum annum* L.), sunflowers (*Helianthus annuus* L.), onions (*Allium cepa* L.), tomatoes (*Solanum lycopersicum* L.)^2–4^, melons (*Cucumis melo* L.), corns (*Zea mays* L.)^5, 6^ and pomegranates (*Punica granatum* L.)^7^. Irrigation with saline water requires the application of extra water to enable the leaching of salts from the root zone in order to prevent their excessive accumulation. The leaching fraction (LF) is defined as the fraction of the amount of water draining beyond the root zone relative to the amount of applied irrigation water^8^. A suitable LF can maintain favorable root zone salinity^9^. By changing the amount of water drains beyond the root zone under a given EC_iw_, the LF adjusts the balance between the soil solution and the EC_iw_^7^.

Yield response curves are often provided, either as a threshold for the electrical conductivity of soil saturated paste extract (EC_e_)^10^, under which no response occurs, combined with the slope of a linear response above that salinity, or as a sigmoidal logistic response model^11^. A reduction in the yield (biomass) as a result of salinity is associated with an equivalent reduction in transpiration^3, 4, 12^. Relative ET can, therefore, be calculated from its proportional relationship to relative yield (biomass). Bhantana and Lazarovitch^7^ showed a 10% reduction in the seasonal ET of two young pomegranates per unit increase of EC_e_ with a threshold of 1 dS m^−1^ using the Maas and Hoffman salinity yield response model. In 2002–2007, the seasonal ET of date palms (*Phoenix dactylifera* L.) was reduced to 50% when the EC_e_ was 4.1–6.36 dS m^−1^ using the van Genuchten and Hoffman salinity yield response model^12^. A sigmoidal logistic response model was also used to calculate the relationship between the ET and EC_iw_ in date palms and leeks (*Allium porrum* L.)^12, 13^. However, whether LFs have an effect on these model parameters is not clear.

In addition to ET, the level of EC_iw_ also affects the salinity of the root zone. In an experiment using bell peppers^2^, the salinity of the drainage water leaving the root zone (EC_dw_) was 1.5–2 times higher than for an EC_iw_ level of 7–9 dS m^−1^. Similarly, in the case of two young pomegranates, a considerably higher EC_dw_ was observed in an EC_iw_ level of 8 dS m^−1 7^. On the whole, there is almost no uptake of salts from the soil by plant roots. The EC_dw_ was increased as a successive accumulation of salts in the soil.

Hot peppers are one of the most popular and widely grown vegetables in the world, and are considered moderately sensitive to salt stress^14–16^. Most studies have been conducted to determine the effect of the EC_iw_ on growth, yield and quality rather than directly determining ET. In addition, a limited number of studies have been conducted to analyze the effects of LFs on drainage water salinity and ET. There is also scant information for nighttime ET under varying EC_iw_ levels and LFs, which accounted for a considerable proportion of the total daily ET and lower crop water productivity. The objective of this study is to combine varying EC_iw_ levels and LFs and assess whether the effect of EC_iw_ on the EC_dw_ and ET can be modified by using LFs.

## Results

### Variations in the EC_dw_ and EC_e_

**Figure 1:**
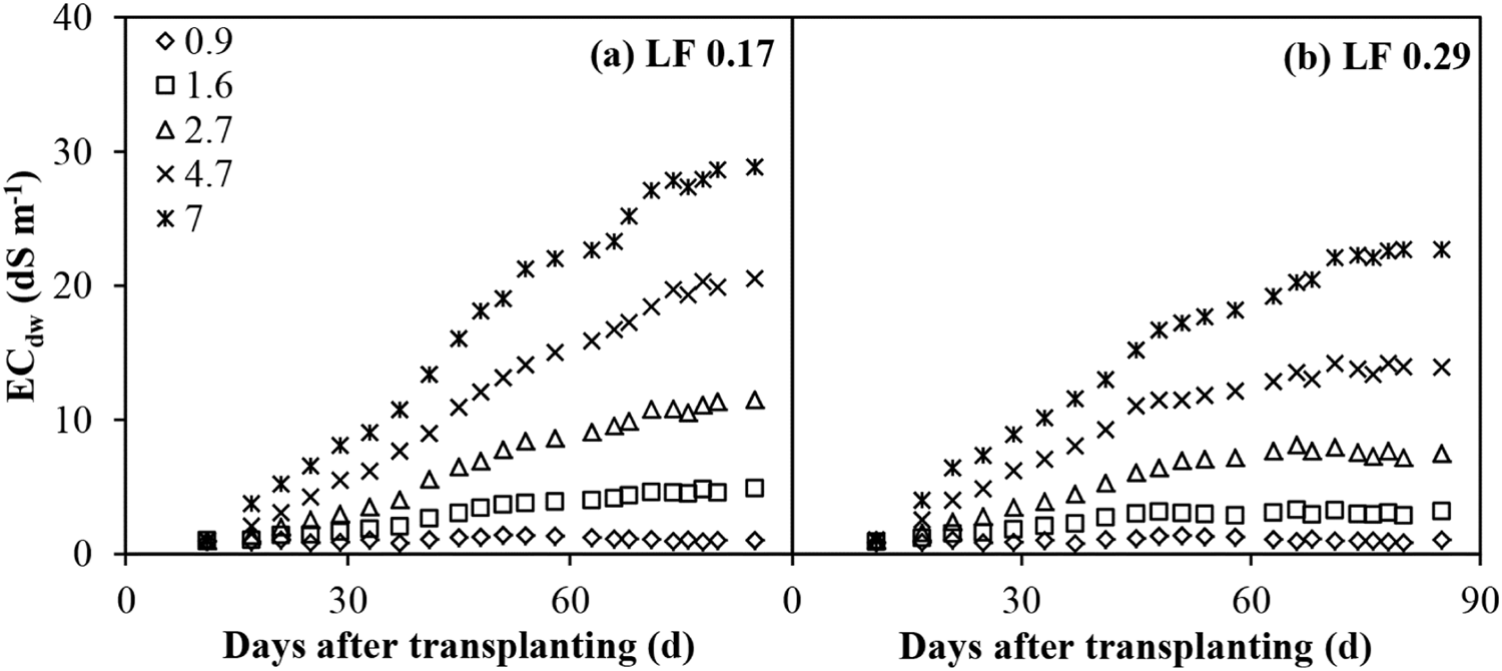
Electrical conductivity of drainage water leaving root zone (EC_dw_) as a function of time and irrigation water salinity under two leaching fractions (LF).

Variations in the EC_dw_ over time are illustrated in Fig. 1. The EC_dw_ became a linear function of EC_iw_ and there were considerable differences among treatments after the second application of saline water (17 days after transplanting (DAT)) for the two LFs. The EC_dw_ reached the EC_iw_ level approximately 25 days after the treatment had been initiated for both LFs. The EC_dw_ of higher salinity levels in the LF of 0.29 reached more or less constant values within 10 weeks. The values of the EC_dw_ for these treatments were 2.0–3.2 times higher than that of the EC_iw_ (Fig. 1). The EC_dw_ of the higher salinity levels in the LF of 0.17 continued to increase throughout the season, however, reaching levels of up to 3.1–4.4 times their corresponding EC_iw_ values.

During 21–36 DAT, the EC_dw_ in the LF of 0.29 was considerably higher than in the LF of 0.17, while the LF had no significant (*P* > 0.05) effect on the EC_dw_ during 1–20 and 37–50 DAT. After 50 DAT, the EC_dw_ in the LF of 0.29 was considerably lower than in the LF of 0.17. At the end of the experiment, the EC_dw_ was 21.4%–35.2% higher in the LF of 0.17 than in the LF of 0.29, except for the EC_iw_ of 0.9 dS m^−1^. There were marked interactions between the EC_iw_ and LF on the EC_dw_ from 54 DAT to the end of the experiment, indicating that the greatest EC_dw_ belonged to the highest EC_iw_ and the LF of 0.17, and the lowest EC_dw_ occurred in the EC_iw_ of 0.9 dS m^−1^ with the LF of 0.29.

The salinity in the soil accumulated when it was irrigated with saline water. The EC_e_ in the different soil layers increased linearly with an increase EC_iw_ level for the two LFs. A reduction in the LF increased the EC_e_ significantly (*P* < 0.001) (Table 1). The average EC_e_ in the LF of 0.17 was 16.0%–33.4% higher than in the LF of 0.29, except for the EC_iw_ of 0.9 dS m^−1^. Salinity was mainly concentrated in the top layer of the soil. The EC_e_ in the 10 cm soil layer was significantly (*P* < 0.001) higher than in the 20 cm soil layer (Table 1). There were significant (*P* < 0.01) interactions between the EC_iw_ and LF in terms of an average EC_e_.

**Table 1:** Electrical conductivity of soil saturated paste extract (EC_e_) in the 10 and 20 cm soil layers under various irrigation water salinity levels (EC_iw_) and leaching fractions (LF) and output of the two–way analysis of variance (ANOVA) for EC_e._

LF	EC_iw_ (dS m^−1^)	EC_e_ (dS m^−1^)		
		10 cm	20 cm	Average
0.17	0.9	1.1 ± 0.08	1.0 ± 0.13	1.1 ± 0.08
	1.6	3.3 ± 0.25	2.6 ± 0.17	2.9 ± 0.15
	2.7	6.3 ± 0.76	4.7 ± 0.11	5.5 ± 0.34
	4.7	9.6 ± 0.95	9.0 ± 0.87	9.3 ± 0.77
	7.0	14.6 ± 2.19	12.4 ± 2.76	13.5 ± 1.98
0.29	0.9	1.2 ± 0.10	1.0 ± 0.13	1.1 ± 0.02
	1.6	2.2 ± 0.42	1.7 ± 0.14	2.0 ± 0.22
	2.7	3.9 ± 0.52	3.4 ± 0.42	3.7 ± 0.46
	4.7	7.6 ± 0.58	5.6 ± 0.48	6.6 ± 0.41
	7.0	13.1 ± 0.26	9.6 ± 0.82	11.3 ± 0.45
**ANOVA**				
Depth	***			
LF		***	***	***
EC_iw_		***	***	***
LF × EC_iw_		NS	*	**

*, ** and *** represent significant differences between means at 0.05, 0.01 and 0.001 level of probability, respectively; NS, non–significant. Each value is mean ± S.D. (n = 4).

## Effects of the EC_iw_ and LF on ET

### Hourly scale

**Figure 2:**
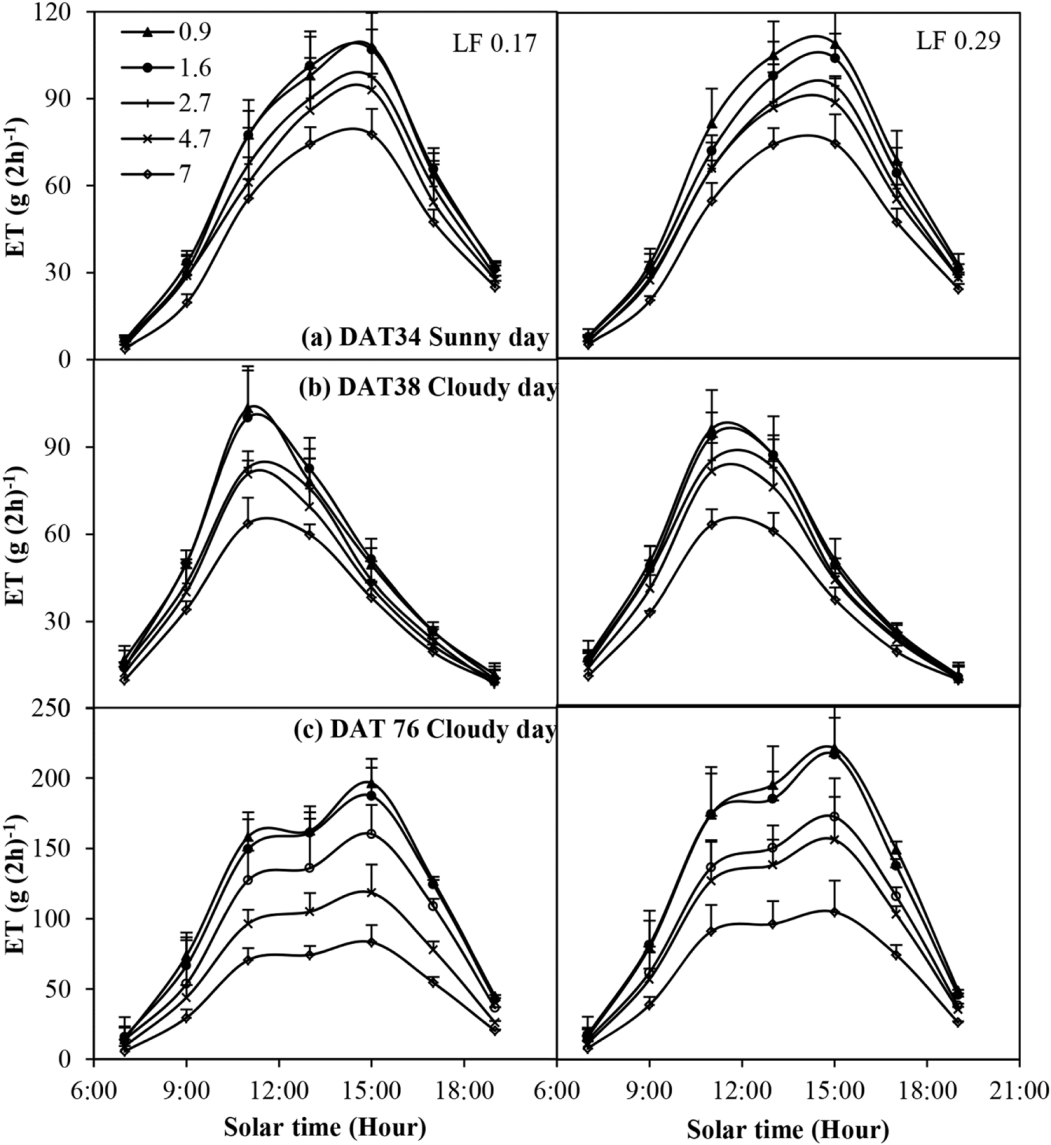
Diurnal variation of evapotranspiration (ET) under various irrigation water salinity levels and leaching fractions (LF). The error bars indicate standard deviation of ET. DAT represents days after transplanting.

Figure 2 shows the diurnal variation of ET every two hours from 7:00 to 19:00 under various EC_iw_ levels for the two LFs at 34, 38 and 76 DAT. An increase in the EC_iw_ levels always linearly decreased the hourly ET, even when there was a low demand for evaporation – at 7:00 and 19:00, for example. Hourly ET was always a function of the EC_iw_ and the slopes of the regression functions were higher at 11:00–15:00 when the demand for evaporation was high. The difference in terms of hourly ET over the different treatments became more marked as time went by (Fig. 2). There was no significant (*P* > 0.05) difference between the two LFs at 34 and 38 DAT with respect to the hourly ET, and no interactive effect between the EC_iw_ and LF at 34, 38 and 76 DAT. At 76 DAT, except for at 7:00, the hourly ET in the LF of 0.29 was considerably higher than in the LF of 0.17, especially when the EC_iw_ were high. The hourly ET of the EC_iw_ level of 4.7 and 7 dS m^−1^ was 20.0%–26.1% higher in the LF of 0.29 than in the LF of 0.17.

### Nighttime ET

**Figure 3:**
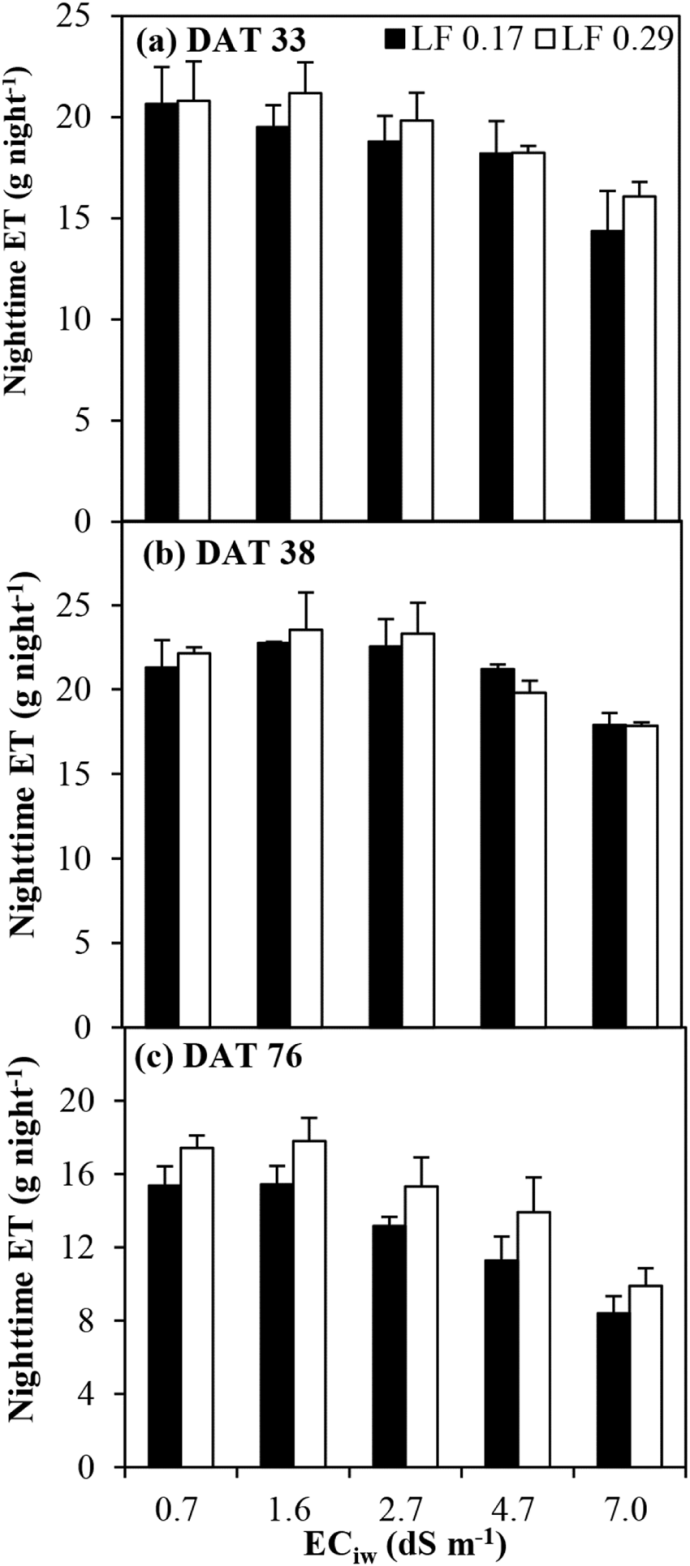
Variation of nighttime evapotranspiration (ET) under various irrigation water salinity levels (EC_iw_) and leaching fractions (LF). The error bars indicate standard deviation of nighttime ET. DAT represents days after transplanting.

Figure 3 shows the variation in terms of nighttime ET under various EC_iw_ levels for the two LFs at 33, 38 and 76 DAT. Nighttime ET accounted for 1.9%–5.6% of the total daily ET. An increase in the EC_iw_ significantly (*P* < 0.001) decreased the nighttime ET (Table 2). The highest nighttime ET was obtained for the EC_iw_ of 0.9 dS m^−1^, and the lowest from the EC_iw_ of 7.0 dS m^−1^. The LF had no significant (*P* < 0.05) effect on nighttime ET at 33 and 38 DAT, while the nighttime ET in the LF of 0.29 was significantly (*P* < 0.001) higher than in the LF of 0.17 at 76 DAT (Table 2). There were no marked interactions between the EC_iw_ and LF in terms of nighttime ET.

**Table 2:** Output of the two–way analysis of variance (ANOVA) for nighttime evapotranspiration (ET) at 33, 38 and 76 days after transplanting (DAT), leaf area, root dry weight and K^+^ and Na^+^ concentrations of hot pepper as affected by various irrigation water salinity levels (EC_iw_) and leaching fractions (LF).

Factor	Nighttime ET (g night^−1^)			Leaf area (m^2^ plant^−1^)	Root dry weight (g plant^−1^)	K^+^ (mg g^−1^ DW)	Na^+^ (mg g^−1^ DW)
	33 DAT	38 DAT	76 DAT				
LF	NS	NS	***	**	NS	NS	**
EC_iw_	***	***	***	***	***	***	***
LF × EC_iw_	NS	NS	NS	NS	NS	NS	NS

*, ** and *** significant differences between means at 0.05, 0.01 and 0.001 level of probability, respectively; NS, non–significant.

### Daily scale and cumulative ET

**Figure 4:**
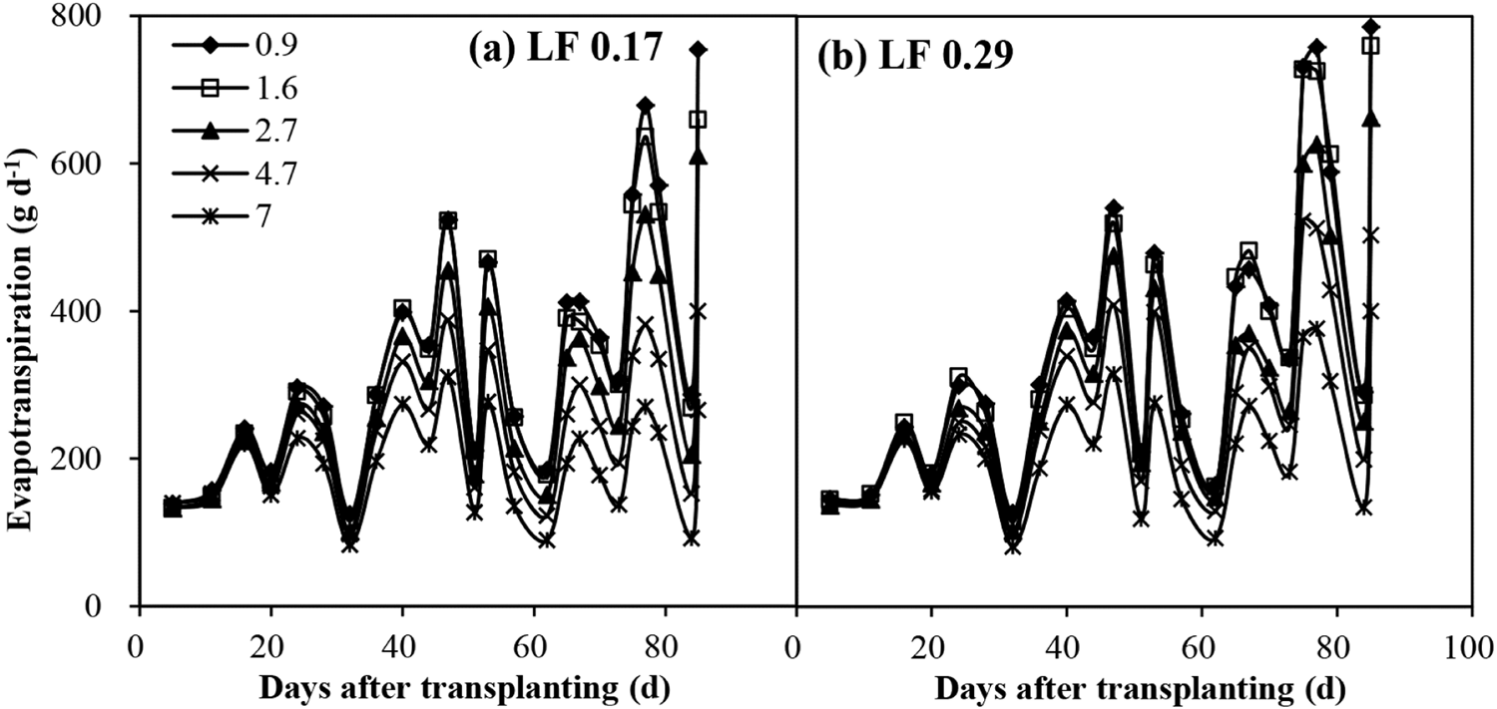
Temporal fluctuation in daily evapotranspiration (ET) throughout the growth season under various irrigation water salinity levels and leaching fractions (LF).

**Figure 5:**
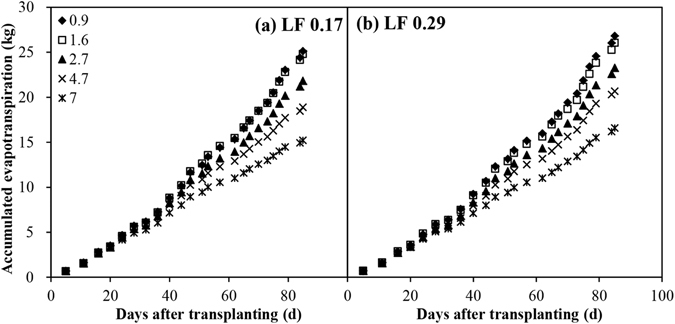
Evolution of cumulative evapotranspiration (ET) throughout the growth season under various irrigation water salinity levels and leaching fractions (LF).

Figures 4 and 5 show the evolution of daily and cumulative ET by aggregating their respective daily ET values under various EC_iw_ levels and LFs. Daily and cumulative ET was nearly identical when all the plants were irrigated using tap water at the beginning of the experiment. A significant (*P* < 0.05) reduction in daily and cumulative ET under varying EC_iw_ levels was observed once treatment commenced (10 DAT). While daily and cumulative ET started to decrease linearly as the EC_iw_ level increased after the second (16 DAT) and third applications (20 DAT) of saline water, respectively, for the both LFs. The absolute value of the regression function slope between daily ET and the EC_iw_ was higher when the demand for evaporation was also high. Salinity reduced the cumulative ET and the extent of the reduction increased with time. Daily and cumulative ET in the LF of 0.29 was considerably higher than in the LF of 0.17 from 65 DAT and 75 DAT, respectively. The difference in terms of cumulative ET between the two LFs increased over time. There was a considerable difference in terms of daily ET between the two LFs on sunny days, especially when the EC_iw_ was higher, for instance the daily ET in the LF of 0.29 was 23.7%–33.3% higher at 75 DAT (a sunny day) and 7.7%–24.8% higher at 73 DAT (a cloudy day) than in the LF of 0.17. Throughout the experiment, there were no marked interactions between the EC_iw_ and LF in terms of the daily and cumulative ET.

### Seasonal ET

Table 3 shows that the seasonal irrigation, amount of drainage water and ET were 18.0–35.9 kg, 3.2–9.7 kg and 15.2–26.8 kg, respectively. An increase in the EC_iw_ level significantly (*P* < 0.001) decreased the seasonal ET. The application of 7.0 dS m^−1^ in the EC_iw_ caused ET to be reduced by 39.5% and 38.1% as compared with the EC_iw_ of 0.9 dS m^−1^ for the two LFs. The seasonal ET increased significantly (*P* < 0.01) because of an increase in the LF, as well as the seasonal irrigation and amount of drainage water. Under varying EC_iw_ levels, the seasonal ET in the LF of 0.29 was 4.8%–8.7% higher than in the LF of 0.17. There were no significant (*P* > 0.05) interactions between the EC_iw_ and LF in terms of the amount of irrigation water and seasonal ET.

**Table 3:** Effects of irrigation water salinity (EC_iw_) and leaching fraction (LF) on seasonal irrigation, amount of drainage water, evapotranspiration (ET) and actual LF using two–way analysis of variance (ANOVA)

LF	EC_iw_ (dS m^−1^)	Irrigation (kg)	Drainage (kg)	ET (kg)	ET (mm)	Actual LF
0.17	0.9	29.2 ± 1.5	4.6 ± 0.3	25.1 ± 1.36	157.0 ± 8.5	0.16 ± 0.001
	1.6	29.0 ± 1.5	4.6 ± 0.3	24.8 ± 1.36	155.0 ± 8.5	0.16 ± 0.007
	2.7	25.6 ± 3.7	4.3 ± 0.5	21.8 ± 3.00	136.3 ± 18.7	0.17 ± 0.007
	4.7	22.2 ± 1.9	3.7 ± 0.2	18.9 ± 1.64	117.9 ± 10.2	0.17 ± 0.007
	7.0	18.0 ± 0.6	3.2 ± 0.1	15.2 ± 0.60	94.9 ± 3.8	0.18 ± 0.004
0.29	0.9	35.9 ± 1.1	9.7 ± 0.3	26.8 ± 0.82	167.5 ± 5.1	0.27 ± 0.004
	1.6	35.1 ± 2.0	9.5 ± 0.5	26.0 ± 2.44	156.7 ± 15.3	0.27 ± 0.005
	2.7	31.2 ± 0.9	8.6 ± 0.2	23.3 ± 0.65	145.3 ± 4.1	0.27 ± 0.002
	4.7	28.0 ± 1.3	7.7 ± 0.3	20.7 ± 0.98	129.1 ± 6.2	0.27 ± 0.003
	7.0	22.3 ± 1.6	6.2 ± 0.3	16.6 ± 1.25	103.6 ± 7.8	0.28 ± 0.008
**ANOVA**						
LF		***	***	**	**	
EC_iw_		***	***	***	***	
LF × EC_iw_		NS	***	NS	NS	

** and *** represent significant differences between means at 0.01 and 0.001 level of probability, respectively; NS, non–significant. Each value is mean ± S.D. (n = 4).

### Evapotranspiration response functions

**Figure 6:**
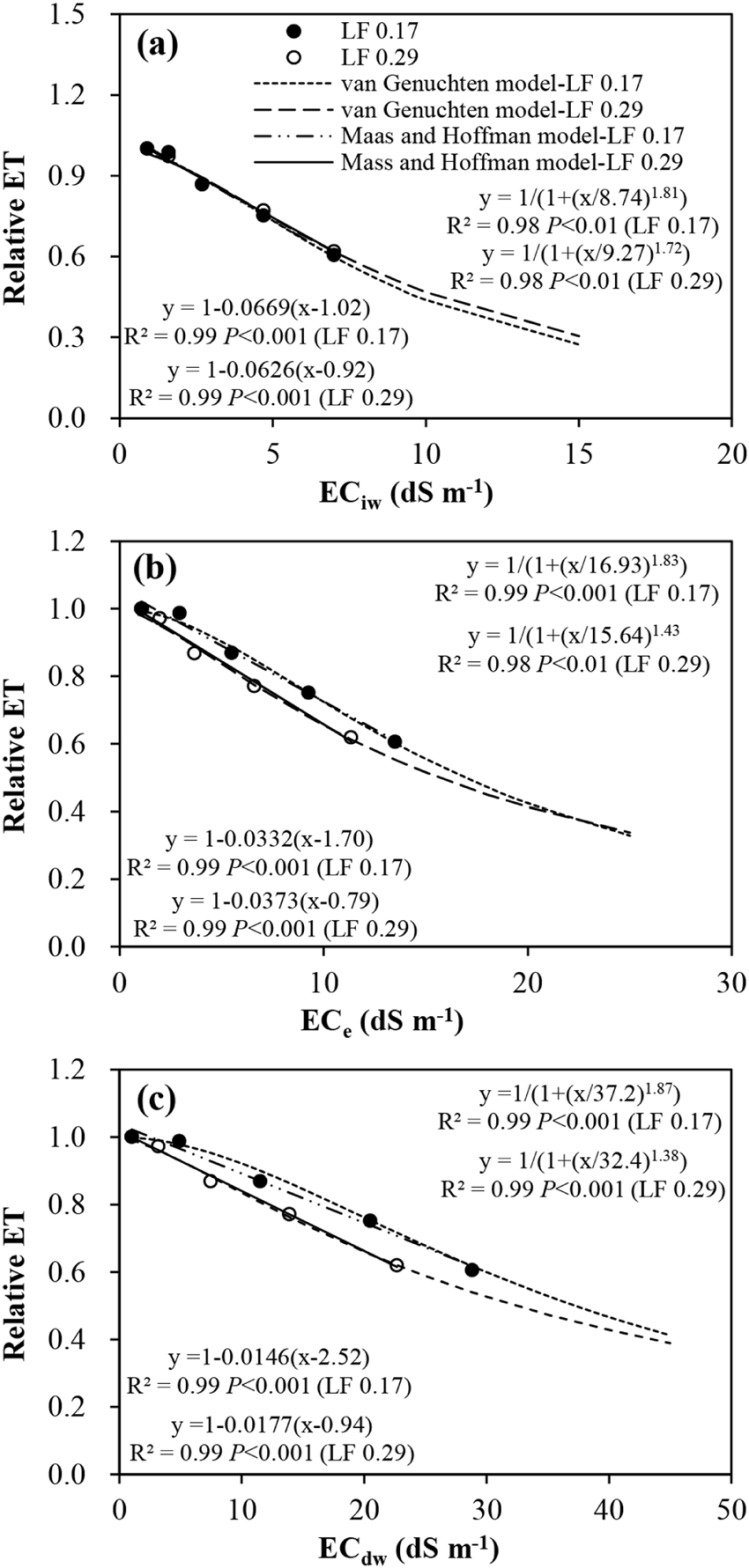
Relative evapotranspiration (ET) as a function of irrigation water salinity (EC_iw_) (**a**), electrical conductivity of soil saturated paste extract (EC_e_) (**b**), and drainage water salinity (EC_dw_) (**c**) for Maas and Hoffman and van Genuchten salinity response models, respectively, under two leaching fractions (LF).

The effect of salinity on ET is further demonstrated by examining ET as a function of EC_iw_, EC_e_ and EC_dw_. Figure 6 shows the relative seasonal ET measured and estimated by the Maas and Hoffman model and van Genuchten and Hoffman model. The LFs had no significant (*P* < 0.05) effect on the parameters of either model. In both models, the relative seasonal ET estimated across the LFs bears a close resemblance to the measured data, with R^2^ ranging from 0.98 to 0.99 (n = 5, *P* < 0.01 or 0.001) (Fig. 6). The estimated values for the EC_iw_, EC_e_ and EC_dw_ threshold given in the Maas and Hoffman model were 0.92–1.02, 0.79–1.70 and 0.94–2.52 dS m^−1^, respectively, for the two LFs, indicating that seasonal ET starts to decrease when the EC_iw_, EC_e_ and EC_dw_ are higher than these values. The slope parameters of the model were 0.0626–0.0669, 0.0332–0.0373 and 0.0146–0.0177 m dS^−1^ respectively for the two LFs (Fig. 6). The curves represented in Fig. 6 also show the b and EC_50_ values calculated on a seasonal basis. The EC_i50_, EC_e50_ and EC_dw50_ were 8.74–9.27, 15.64–16.93 and 32.4–37.2 dS m^−1^, respectively, and the values of b were 1.72–1.81, 1.43–1.83 and 1.38–1.87, respectively for the two LFs.

### Leaf area, root dry weight and K^+^ and Na^+^ concentrations

**Figure 7:**
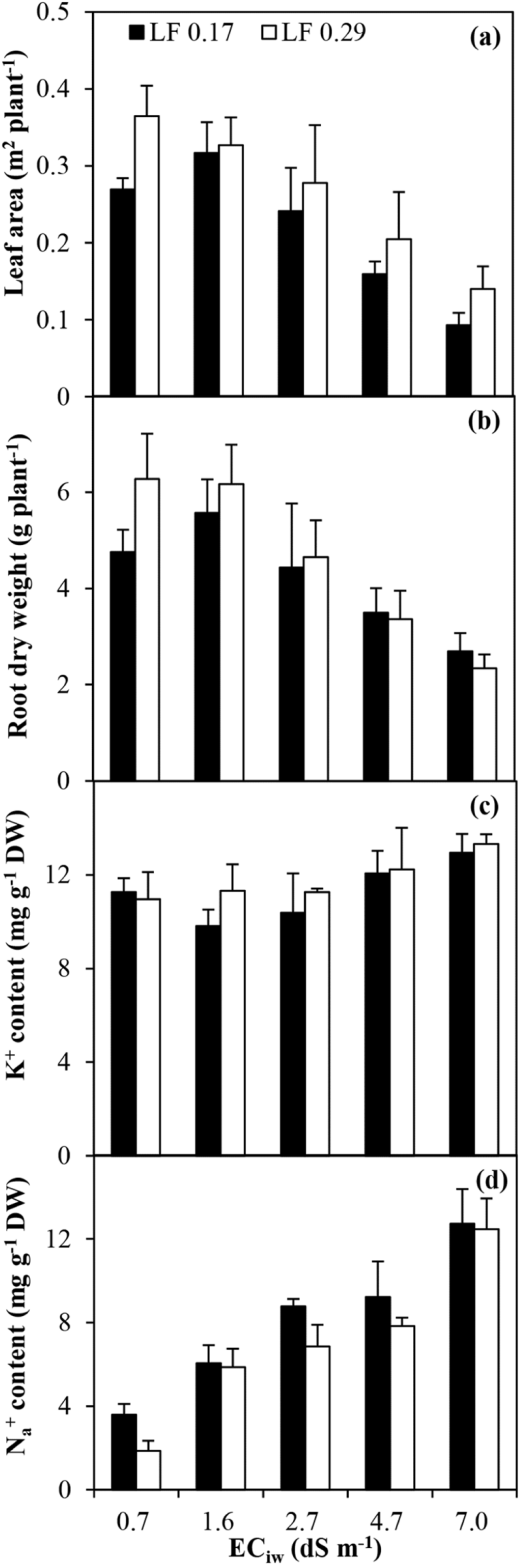
Leaf area (**a**), root dry weight (**b**) and K^+^ (**c**) and Na^+^ (**d**) concentrations of roots under various irrigation water salinity levels (EC_iw_) and leaching fractions (LF). The error bars indicate standard deviation.

Leaf area and root dry weight values under various EC_iw_ and LFs are shown in Fig. 7. The leaf area and root dry weight showed a pronounced reduction with an increase in the EC_iw_ level, especially when the EC_iw_ level was high. Compared with the EC_iw_ of 0.9 dS m^−1^, the leaf area and root dry weight in the EC_iw_ of 7.0 dS m^−1^ decreased by 61.6%–65.5% and 43.3%–62.8% respectively for the two LFs. The high LF significantly (*P* < 0.01) increased the leaf area (Table 2). Neither LF had any effect on root dry weight, however. There were no marked interactions between the EC_iw_ and LF on the leaf area and root dry weight.

An increase in the EC_iw_ led to an increase in the K^+^ and Na^+^ concentrations in the roots (Fig. 7). The Na^+^ concentration in the roots decreased significantly (*P* < 0.01) owing to an increase in the LF, while the LF did not affect the K^+^ concentration (Table 2).

## Discussion

As we have shown, the EC_iw_ and LFs have a strong effect on the EC_dw_ and EC_e_. Non–saline soil was used in this study. After being irrigated with saline water several times, the salt accumulated rapidly in the soil in the high LF as a result of the introduction of more saline water; this is reflected by the considerably higher EC_dw_ in the LF of 0.29 than in the LF of 0.17 during 21–36 DAT. When salt began to accumulate in the soil, more drained water in the higher LF resulted in more salt being leached from the root zone, so that the salt that had accumulated in the higher LF became lower than in the lower LF, as is reflected by the difference in the EC_dw_ after 50 DAT (Fig. 1). At the end of the experiment, the EC_dw_ and average EC_e_ in the LF of 0.17 were 21.4%–35.2% and 16.0%–33.4% higher than in the LF of 0.29 respectively, except for the EC_iw_ of 0.9 dS m^−1^ (Table 1). In an experiment using wheat and barley, the EC_e_ in an LF of 0.2 decreased by 49.7%–65.2% when compared with that in an LF of 0.5^17^. The salinity changes in the soil and drainage water for different LFs demonstrate that the addition of water, in excess of that required by hot peppers, could be applied to ensure leaching, thereby controling soil salinity.

The EC_dw_ and EC_e_ increased linearly as the EC_iw_ increased and the extent of the increment increased with time. EC_dw_ that was 1.5 to 2–fold higher than the EC_iw_ was reported for an EC_iw_ of 7–9 dS m^−1^ in an experiment using bell peppers^2^. Bhantana and Lazarovitch^7^ found that EC_dw_ was more than 5 times higher than the EC_iw_ for an EC_iw_ of 8 dS m^−1^ during the peak season. At the end of the experiment covered in this paper, the EC_dw_ was 3.2–4.4 times higher than the EC_iw_ when the EC_iw_ was at 7.0 dS m^−1^ for both LFs. The higher EC_dw_ in the LF of 0.17 did not reach its steady state with a target value of 42 dS m^−1^ according to the concept of LF for steady state conditions, with no precipitation or dissolution and good drainage, i.e., LF = V_d_ / V_i_ = EC_iw_ / EC_dw_^18^, where V_d_ and V_i_ represent the drainage and amount of irrigation water.

The EC_e_ in the 10 cm soil layer was approximately 1.25 times higher than in the 20 cm soil layer in this study. This is because salts always move with water when it evaporates, indicating that salts tend to accumulate in the upper part of the root zone^19, 20^.

The ET of the hot peppers decreased considerably as a result of an increase in the EC_iw_. The hourly ET during the daytime linearly decreased even in the morning when solar radiation was lower (Fig. 2). Similar results were also recorded at night (Fig. 3; Table 2). This means that salinity always affects ET. Root water uptake is mainly driven by the soil’s osmotic and matric potential, which controls their respective symplastic and apoplastic pathways independently^7, 21^. The effect of salinity on ET has generally been assumed to reduce water availability by reducing the osmotic potential^18, 22, 23^. The osmotic stress reduces the free energy of water and causes a plant to spend more biological energy in taking up water from the soil solution, thus causing a reduction in transpiration and ET^24–27^. In addition, the excessive absorption of Na^+^ by the roots in the high EC_iw_ is another reason limiting ET (Fig. 7; Table 2). Salinity also has an adverse effect on the leaf area and root of the plant (Fig. 7; Table 2), limiting the root water uptake rate, which in turn decreases transpiration and ET^6, 28^.

High LF can control soil salinity and in turn increase ET. In this study, the seasonal ET increased significantly (*P* < 0.01) by 4.8%–8.7% in the high LF (Table 3). The possible reasons for this result are as follows: (1) a low LF increases soil salinity (Table 1), thus reducing water availability and causing a reduction in transpiration and ET; (2) when the LF is low, the reduced leaf area (Fig. 7; Table 2) contributes to a reduction in transpiration and ET; (3) a low LF causes the root to absorb more Na^+^ (Table 2) which limites transpiration and ET; and (4) the roots have no effect on the reduction of ET because the LF has no effect on root dry weight (Table 2).

However, the LF did not have an effect on ET once treatment commenced. There were no significant (*P* > 0.05) differences between the two LFs at 34 and 38 DAT in terms of hourly ET (Fig. 2). There was a considerable difference between the two LFs with respect to the daily and cumulative ET from 65 and 75 DAT, respectively. Interestingly, the effect of the LF on ET and EC_dw_ was not synchronous, as described above. The response time of ET to LF was delayed by 15–25 days when compared with that of the EC_dw_. The possible reason for this is that osmotic stress needs time to affect plant growth (e.g. leaf area), which in turn affects plant transpiration and ET.

## Conclusions

In summary, the present study demonstrates that the EC_iw_ always decreases ET considerably in pot–grown hot peppers, even when there is a lower evaporation demand. The Maas and Hoffman and van Genuchten and Hoffman models fitted the measured relative seasonal ET of our EC_iw_ treatments and the LFs had no effect on model parameters. The EC_dw_ and EC_e_ increased linearly with an increase in the EC_iw_, with soil salinity mainly being concentrated in the 0–10 cm soil layer. The effect of the EC_iw_ on the ET, EC_dw_ and EC_e_ was modified by the LF. The EC_dw_ in the LF of 0.29 was considerably higher during 21–36 DAT and considerably lower after 50 DAT, than in the LF of 0.17. The LF had a marked effect on the daily ET from DAT 65. We can therefore conclude that the effect of the LF on the ET and EC_dw_ was not synchronous. Overall, the EC_dw_ and EC_e_ markedly increased, while the seasonal ET decreased because of an increase in the EC_iw_ and a decrease in the LF. The outcome of this study, together with available information on plant responses to constant salinity and LF, should provide valuable information for agricultural water management when saline water irrigation is used.

## Materials and Methods

### Experimental site and plant materials

The experiment was conducted in a rain shelter between April and July 2015 at the Agro–Meteorology Research Station at Nanjing University of Information Science and Technology, located in Nanjing City, Jiangsu Province in Eastern China (32.2° N, 118.7° E, 14.4 m above sea level). Plastic pots with holes in the bottom for drainage were used. Each pot had a diameter of 26 cm at the top and 22 cm at the bottom and a height of 27 cm. The soil was sieved through a 5 mm screen to remove large particles and dry soil aggregates. Each pot was filled with 11 kg of air–dried soil with a sandy loam texture consisting of sand (75.7%), silt (20.4%) and clay (3.9%). The bulk density of the soil was 1.47 g cm^−3^, the field water capacity was 0.27 (cm^3^ cm^−3^), the wilting point was 0.04 (cm^3^ cm^−3^), the electrical conductivity (EC; paste) and pH of the soil were 0.59 dS m^−1^ and 7.4, respectively.

The hot pepper plants (Bocuiwang cultivar) were transplanted to plastic pots (one plant per pot) on April 28, 2015. Before the transplanting took place, the pots were saturated with tap water. Until the plants were established, they were irrigated using tap water. After they were established (10 DAT), saline water treatments under different LFs were started. The distances between the hot pepper pots and within rows were 40 cm.

### Experimental design and measurements

The experiment was arranged in a completely randomized block design with four replications per treatment. Five levels of EC_iw_ (i.e. 0.9, 1.6, 2.7, 4.7 and 7.0 dS m^−1^) and two LFs (i.e. 0.17 and 0.29) were included as factors. Salinity was increased by adding 1:1 milli equivalent concentrations of NaCl and CaCl_2_ to fertilizers. Fertilizers (half strength Hoagland solution) were provided in constant concentrations with the irrigation water which contained^29^: 2.0 mM Ca(NO_3_)_2_ × 4H_2_O, 2.0 mM KNO_3_, 0.5 mM NH_4_NO_3_, 0.5 mM MgSO_4_ × 7H_2_O, 0.25 mM KH_2_PO_4_, 40 uM Fe–EDTA, 25 uM H_3_BO_3_, 2.0 uM MnCl_2_ × 4H_2_O, 2.0 uM ZnSO_4_ × 7H_2_O, 0.5 uM CuSO_4_ × 5H_2_O, 50 uM KCl, 0.075 uM (NH_4_)_6_Mo_7_O_24_ × 4H_2_O, 0.15 uM CoCl_2_ × 6H_2_O. Fertilizers added an EC of 0.9 dS m^−1^ to the irrigation water for all treatments.

Evapotranspiration (ET, g) was calculated by using the following water balance method:

1 $${\text{ET}=W}_{{\rm{n}}}-{\rm{W}}{}_{n+1}+{({\rm{I}}}_{{\rm{n}}}-{{\rm{D}}}_{{\rm{n}}})\times {\rm{\rho }}$$

where W_n_ and W_n+1_ are the weights of pot, plant and soil before the n^th^ and (n + 1)^th^ irrigation (g). I_n_ and D_n_ are the amounts of applied irrigation and drainage water (L) in the n^th^ irrigation, respectively and *ρ* is the water bulk density (1000 g L^−1^). The amount of applied irrigation water (AW) was 120% and 140% of the ET, which resulted in an LF of 0.17 and 0.29 in accordance with the equation proposed by Letey *et al*.^8^:

2 $$\frac{{\rm{AW}}}{{\rm{ET}}}=\frac{1}{{\rm{LF}}}$$

Each pot was weighed just before each irrigation event. Throughout the experiment, the plants were irrigated at 2–5 day intervals at 16:00–17:00. A glass bottle was placed underneath each pot in order to collect the drainage water. The volume and salinity of the collected drainage water were measured after each irrigation event and the actual LF and crop ET were calculated. The application of an LF of 0.17 and 0.29 resulted in an average actual LF of 0.17 and 0.27 (Table 3). The hourly ET was measured every two hours at 34, 38, 76 DAT from 7:00 to 19:00 by weighing. The nighttime ET was measured between sunset (at 19:00) and sunrise (at 5:00) at 33, 38 and 76 DAT. The EC_dw_ was measured after each irrigation event, and the EC_e_ in the 10 and 20 cm soil layers was measured at the end of the experiment by a dual channel pH/mV/Ion/Conductivity benchtop meter (MP522, Shanghai San–Xin Instrumentation Inc., China). The leaf length and maximum leaf width were also measured at the end of the experiment. The leaf area was calculated by summing the lamina length × maximum width of each leaf and multiplied by a factor of 0.54 (our measurement). The roots of each plant were washed in fresh water and dried in an oven at 70 °C to obtain a constant dry weight. The dried roots were then ground into powder. The powdered plant samples were digested by concentrated HNO_3_ heated using a heating block and finally dissolved in 5% (v/v) high–purity HNO_3_. The concentrations of Na^+^ and K^+^ were determined by Inductively Coupled Plasma–Optical Emission Spectrometry (ICP–OES, Perkin Elmer Optima 8000).

### Evapotranspiration response functions

In this study, the relative seasonal ET (ET / ET_m_) data were fitted to the yield reduction model because a reduction in yield as a result of salinity is associated with an equivalent reduction in ET^3, 12^. One is a two–piece linear response function proposed by Maas and Hoffman^10^:

3 $$\frac{{\rm{ET}}}{{{\rm{ET}}}_{{\rm{m}}}}=\{\begin{array}{ll}1 & 0\le {{\rm{EC}}}_{{\rm{e}}}\le {{\rm{EC}}}_{{\rm{t}}}\\ 1-{\rm{b}}({{\rm{EC}}}_{{\rm{e}}}-{{\rm{EC}}}_{{\rm{t}}}) & {{\rm{EC}}}_{{\rm{t}}} < {{\rm{EC}}}_{{\rm{e}}} < {{\rm{EC}}}_{{\rm{o}}}\\ 0 & {{\rm{EC}}}_{{\rm{e}}} > {{\rm{EC}}}_{{\rm{o}}}\end{array}$$

where ET_m_ is the maximum ET, which appeared mainly in an EC_iw_ of 0.9, and 1.6 dS m^−1^, EC_t_ (dS m^−1^) is the threshold electrical conductivity, and b (m dS^−1^) is the slope parameter, indicating the percentage of ET loss per unit increase in the EC_e_ beyond the threshold value, and EC_o_ is the root zone salinity beyond which the yield is zero.

There is another non–linear yield reduction model that is more accurate in terms of describing the sigmoidal growth response of plants to salinity^11^. It is an initial plateau and subsequent decreasing section that better accounts for higher salinity:

4 $$\frac{{\rm{E}}{\rm{T}}}{{{\rm{E}}{\rm{T}}}_{{\rm{m}}}}=\frac{1}{{1+(\text{EC}}_{{\rm{e}}}/\text{EC}{}_{{\rm{e}}50}{)}^{{\rm{b}}}}$$

where EC_e50_ represents the EC_e_ when ET/ET_m_ = 0.5, and b is an empirical, presumably crop, soil and climate–specific dimensionless parameter.

We applied these two models to assess the effect of salinity on ET. We also used EC_iw_ and EC_dw_ instead of EC_e_ in equations (3) and (4) to assess the EC_iw_ and EC_dw_ on relative seasonal ET.

### Statistical analysis

Statistical analyses were performed using an SPSS software package (Version 21.0, IBM Corp., Armonk, NY). Two–way analyses of variance (ANOVA; SPSS) were made to determine the effects of the EC_iw_ and LF on measured EC_dw_, EC_e_, and hourly, nighttime, daily, cumulative and seasonal ET, leaf area, root dry weight, and Na^+^ and K^+^ concentrations.
